# The linked nail/plate construct for the management of distal femur fractures in the elderly

**DOI:** 10.1051/sicotj/2024016

**Published:** 2024-05-30

**Authors:** Georgios Saraglis, Anwar Khan, Amit Sharma, Sagar Pyakurel, Sayed Fazal Elahi Rabbani, Mohamed Shawky Abdelhamid Arafa

**Affiliations:** Department of Trauma and Orthopaedics, Bedfordshire NHS Foundation Trust, Bedford Hospital South Wing, Kempston Road Bedford MK429DJ United Kingdom

**Keywords:** Distal femur fractures, Nail/plate fixation, Double fixation, Augmented fixation

## Abstract

*Background*: Distal femoral fractures represent a challenging injury, with many different factors such as the method of fixation, complexity of fracture pattern, and patient co-morbidities affecting the outcome. Lots of surgical treatment options have been described, but recently double construct fixation, using a nail/plate combination, has received lots of attention, a technique that leads to faster weight-bearing, low risk of metalwork failure, and non-union. The purpose of this study was to investigate the effectiveness of the linked nail/plate construct in the management of complex distal femur fractures and to investigate if the above technique leads to faster recovery and earlier radiographic union. *Materials and methods*: In total 15 cases were included in the study, that underwent a combined nail/plate construct for a distal femur fracture between January 2021 and December 2022. Only cases with a linked nail/plate construct were included, with a minimum follow-up of 1 year. Open femur fractures, single implant fixation cases, and revision procedures were excluded. *Results*: In this cohort study, 11 cases were periprosthetic distal femur features, and 4 cases were distal femur fractures around a native knee joint. The mean age group was 74 years, 86.6% of the patients had a BMI > 25 and the mean time to fracture union was 24 weeks (range from 20 to 26 weeks). All cases healed uneventfully and the complication rate was 6.6%, including 1 case of superficial infection which resolved completely with oral antibiotics. *Conclusion*: The increasing age population, the complexity of distal femoral fractures along with the increasing physiological demands of the elderly population, drive the need for double fixation constructs that allow early mobilization and enhance fracture stability. In our study, the linked nail/plate construct seems to provide adequate stability and excellent union rates (100%) with no associated increased risk of complications.

## Introduction

The management of distal femur fractures in the elderly has always been challenging, as commonly includes geriatric patients with multiple co-morbidities and complex fracture configurations. With high mortality rates (90-day mortality rate of 11% and 1-year mortality rate of 21%, respectively) [1], the management of these complex injuries has drawn lots of attention, and the optimal surgical technique for these injuries still remains unclear.

A new novel technique, the nail and plate construct (NP), has been introduced, providing patients with enhanced stability and, stronger construct, allowing immediate weight-bearing [2]. The use of a single lateral plate (LP) or a single retrograde intramedullary nailing device (rIMN) has raised concerns over non-union rates and metalwork fatigue and failure, while these constructs do not always allow for full weight bearing immediately postoperatively [3, 4]. Rates of non-union are up to 19% whereas the implant failure rate can be as high as 20% [5].

The exact mechanism of failure of the single implant fixation method (rIMN or LP) still remains unclear. Patient characteristics, degree of comminution, possible involvement of the prostheses, or the presence of critical bone defects have all been described as potential factors to failure [6, 7]. Occasionally, the presence of severe metaphyseal comminution, loss of medial cortical continuity, or the presence of interarticular extension of the fracture site can increase the challenges for optimal surgical fixation methods. Biomechanically, eccentric loading with inadequate cortical support could potentially lead to varus collapse, leading to critical metalwork failure and non-union [8].

Cases of metalwork failure and subsequent nonunion have been described for both rIMN and LP fixation methods [8]. As a rule of thumb, distal femur fractures with inadequate bone stock for sufficient nail anchorage are being treated with LP constructs with limited or restricted weight bearing post-operatively whereas even with the single rIMN constructs not all patients are allowed to fully weight bearing post operatively [9].

In an attempt to overcome the above challenges and in order to provide the option of early weight bearing postoperatively, even for complex injuries, the nail plate (NP) has been introduced [10]. The NP construct includes the synergic use of a retrograde intramedullary nail (rIMN) and a distal lateral plate (LP), in order to maximize the stability of the construct leading to a sturdy fixation.

Despite several studies, focusing on the common complications of single implant constructs for the management of complex distal femur fractures, only a few studies focus on new hybrid techniques that can provide an adequate fixation method addressing the challenges that the complex distal femur fractures pose to the orthopedic surgeon [10, 11].

In this cohort study, we present a new novel technique that has been used in our institution with excellent results. The technique includes the combined use of a retrograde intramedullary femoral nail and distal lateral femoral locking plate with the two implants linked together using an interlocking compression bolt (condylar screw). Apart from excellent union rates and early mobilization, this technique seems to address the common challenges of distal femur fractures, utilizing the synergic use of two different implants, providing enhanced stability and rigid fixation even in complex fractures with osteoporotic bone stock.

## Materials and methods

This was a retrospective cohort study focusing on the clinical and radiographic performance of patients that underwent a linked retrograde intramedullary femoral nailing (T2 retrograde IM nailing, Stryker, Kalamazoo, Minnesota, USA)/distal femoral locking plate (AxSOS 3 distal lateral femur plate, Stryker, Kalamazoo, Minnesota, USA).

The analysis was conducted on 15 patients of all ages (mean age 74 years) that underwent a combined nail/plate fixation for distal femur fractures in a single institution in the UK between January 2021 and December 2022, with a minimum radiographic follow-up of 1 year (Table 1).

**Table 1:** Patient demographics.

	Periprosthetic distal femur fractures	Native distal femur fractures	Total
Number of cases	11 cases	4 cases	15 cases
Gender: male/female	4M/7F	2M/2F	15 cases
Mean age	75 years	72 years	74 years
Mean time to union	21–26 weeks	20–23 weeks	24 weeks
Mean BMI	50%, BMI > 30	40%, BMI > 30	86.6%, BMI > 25

M: Males; F: Females; BMI: Body Mass Index.

Only cases that underwent a linked nail/plate construct were included in the above study and included cases of periprosthetic distal femur fractures, complex distal femur fractures, and osteoporotic distal femur fractures of the elderly. All patients received the same type of fixation (linked nail/plate construct) using the same implants and in all cases, patients were allowed to mobilize full weight bearing immediately postoperatively.

Patient demographics, mean time to radiographic union, type of implants used, and complications were included in the statistical analysis. Exclusion criteria included: open femur fractures, distal femur fractures treated with single implant and revision procedures.

For all cases, a pre-operative lower limb multi-disciplinary team (MDT) meeting was performed, and the indication for nail/plate construct fixation was documented in the patient’s records (obesity, complex fracture pattern including medial cortex comminution, osteoporosis/severe osteopenia, presence of a segmental defect, etc) and all cases were performed by 2 experienced orthopedic trauma surgeons familiar with nail/plate technique.

The primary aim of this study was to investigate the effectiveness of the linked nail/plate construct in the management of complex distal femur fractures and to investigate if the above technique leads to faster recovery and higher radiographic union. A secondary aim was to investigate if the linked nail/plate construct was associated with a higher complication rate in comparison to single construct techniques. A comparison of the study results was performed with the cohort study from 2021, in which Passias et al. [12] reviewed 97 distal femur fractures in a level-1 trauma center, including both single construct and NP construct fixation techniques.

## Surgical technique

The patient is positioned supine on a radiolucent table. The affected lower extremity and hip region are freely draped and the knee is placed over a sterile bolster, maintaining approximately 30° of flexion.

Following standard preparation and draping, the procedure begins with a 3 cm midline incision extending from the inferior pole of the patella to the tibia tubercle. The correct entry point for the guide wire is confirmed with fluoroscopy, followed by a proximal entry reamer using a retrograde protection sleeve. The long femoral guide wire is inserted and the appropriate length of the femoral nail is selected.

As a rule of thumb, the length of the retrograde nail should be at the level of the lesser trochanter in order to provide adequate support and splinting of the entire femur. Following standard femoral preparation and canal reaming the retrograde nail is inserted. At this stage, a separate direct lateral approach to the femur is performed and the fracture site is visualized under direct vision.

An adequate-length distal femoral locking plate is selected and the provisional position of the plate is confirmed under fluoroscopy. The final position of the lateral plate is maintained using Kirschner wires and reduction clamps. Once the optimal position of the plate is achieved the first screw option is the condylar screw (compression bolt) of the retrograde nail.

Using the “perfect circle” technique and under fluoroscopy, the condylar screw is inserted through the rIMN jig and through the distal lateral locking plate, linking the two implants together. This is followed by separate distal interlocking screws for the rIMN and several distal locking screws for the lateral plate. The proximal screw holes of the lateral plate are filled with unicortical screws, while occasionally circlage wiring can be added for maximum stability.

Finally, the rIMN is secured proximally by inserting one anteroposterior screw at the level of the lesser trochanter. Postoperatively, all patients are allowed to mobilize full weight bearing using a knee brace for additional support for four weeks and regular radiographic follow-up is arranged until evidence of radiographic union.

## Results

In this cohort study, 15 cases were included that presented to our institution with a distal femur fracture and all cases were treated using the same implants (linked implants using a condylar screw).The retrograde nail used was the Stryker T2 femoral supracondylar nail and the plate was the Stryker AxSOS distal lateral femur plate.

**Figure 1:**
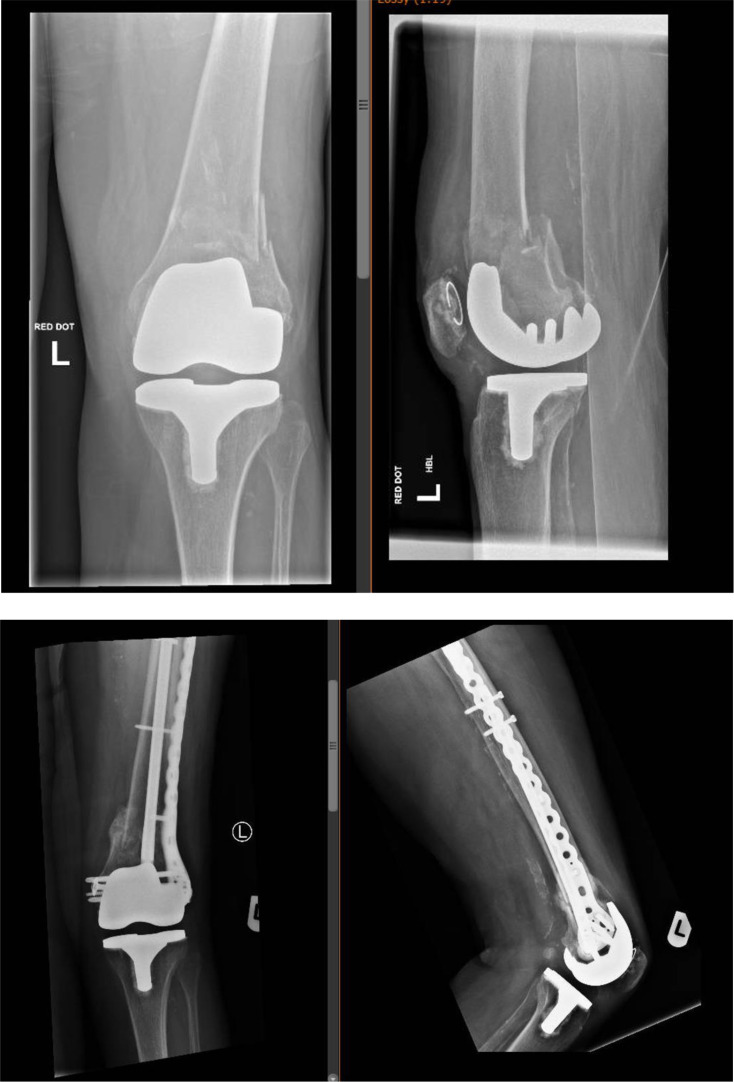
Pre-operative and post-operative radiographs (fracture union) of linked nail/plate construct of distal femur periprosthetic fracture.

**Figure 2:**
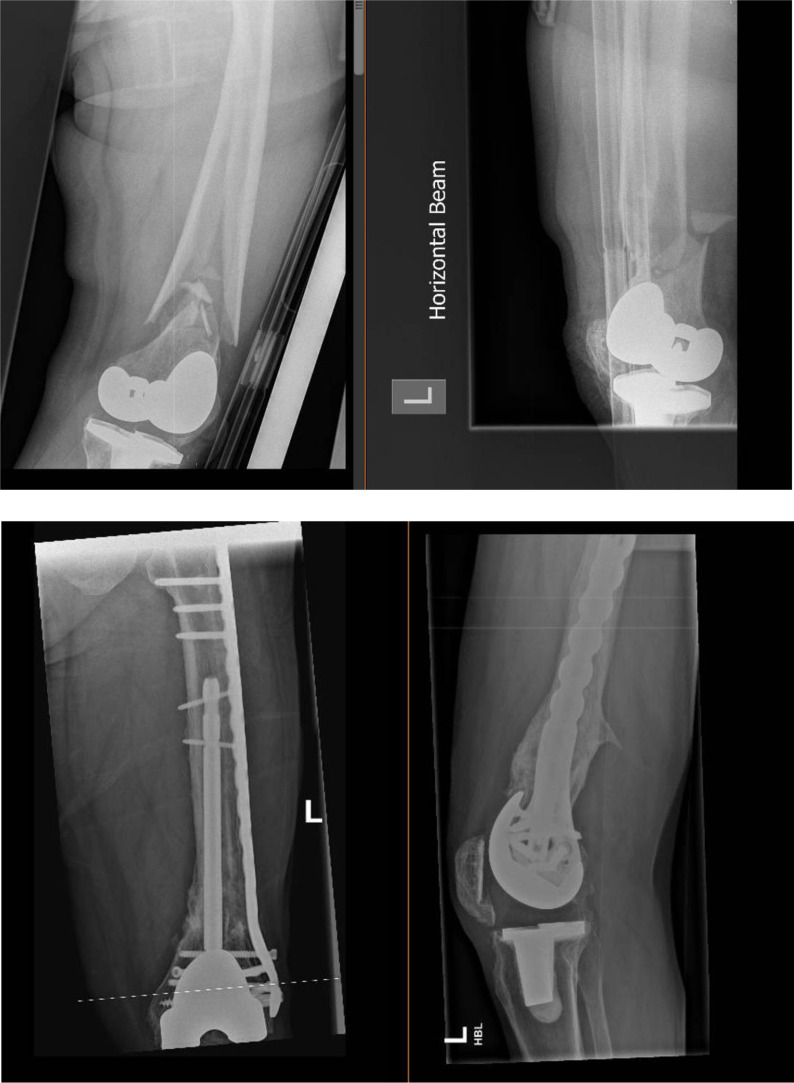
Pre-operative and post-operative radiographs (fracture union) of linked nail/plate construct of distal femur periprosthetic fracture (combination of short rIMN/long bridging LP).

**Figure 3:**
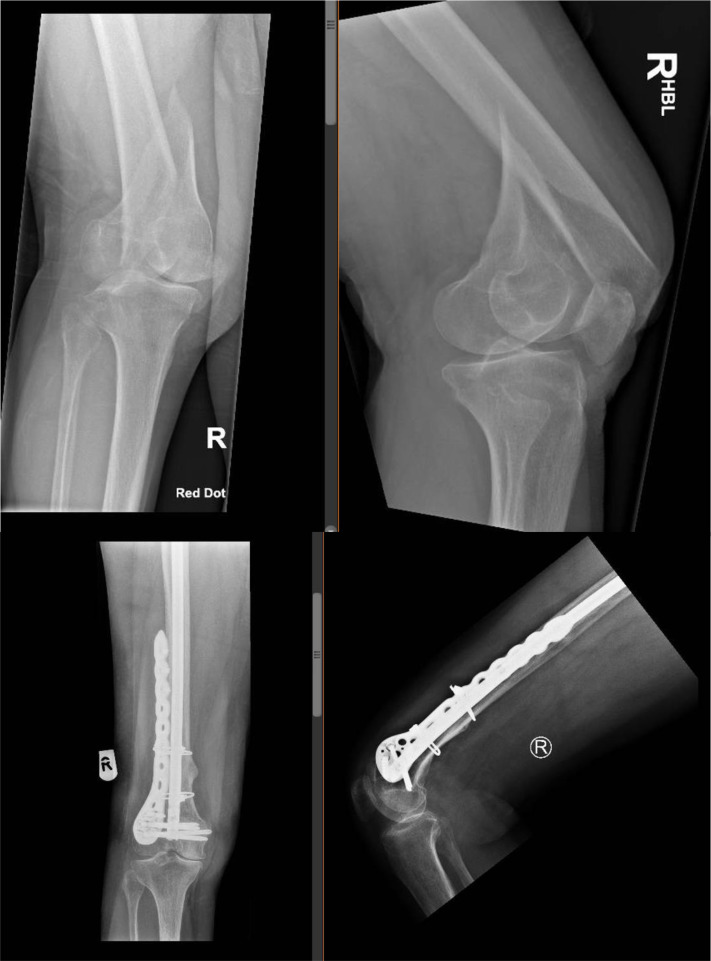
Pre-operative and post-operative radiographs of distal femur fracture (native joint) treated with linked nail/plate construct.

The mean age group in this cohort of patients was 74 years, 13 cases were patients with high BMI > 25 and severe osteoporosis, 11 cases were periprosthetic distal femur fractures around a well-fixed knee replacement (Figures 1 and 2) and 4 cases included distal femur fractures around the native knee joint (Figure 3).

The mean time to fracture union was 24 weeks (range from 20 to 26 weeks), and all cases healed uneventfully. The fracture union was defined both radiologically, with the presence of radiographic callus formation in 3/4 cortices, and clinically with the patients being able to mobilize with no pain during the follow-up assessment. Similarly to the study by Passias et al. [12], where the NP construct group achieved excellent radiographic union rates, in our study the radiographic union was 100% vs. 69% of the union rate of the single construct fixation group (rIMN or LP) in the study by Passias et al.

The complication rate was 6.6% and included one case of superficial wound infection developed two weeks postoperatively, treated successfully with a course of oral antibiotics for one week, with complete resolution of the infection and no further surgical intervention. Comparison of the complication rate of our study with the overall complication rate by Passias et al., comparing single construct vs. nail/plate construct cases, did not reveal any statistically significant differences (6.6% vs. 5.6% for single implant fixation group by Passias et al.).

## Discussion

Distal femur fractures can be a great challenge, even for the most experienced trauma surgeons, and could pose a significant challenge to stable fixation. In the case of periprosthetic fractures, the presence of an underlying prosthesis, the lack of anatomical landmarks, and the underlying osteoporotic bone can make the accuracy of anatomical reduction challenging while the blood supply around the knee may also be impaired [11]. Traditionally, distal femur fractures are treated using a rIMN or a distal lateral femur plate depending on fracture configuration, patient factors, and surgeon preference. Hardware failure though, with single implant fixation has been well-described in several studies [11] with the eccentric load leading to varus collapse being the most well described method of failure [11]. To overcome this common complication, several surgical techniques have been developed focusing on the double fixation constructs allowing early mobilization.

There were some limitations in the above study. This was a retrospective nonrandomized study focusing on a single surgical technique. There was no direct comparison between different surgical technique groups and the comparisons were conducted against a literature cohort study (Passias et al., level-1 trauma center study) [12]. Additionally, the number of cases included can be considered low (15 cases) but in comparison to other similar studies this is among the very few studies with a relatively high number of cases (>10 cases) and among the very limited studies focusing on the linked nail/plate construct.

The nail/plate construct has been described by many authors with excellent outcomes [13, 14]. In the study of Kanabur et al. [14], all patients who received a nail/plate fixation had radiographic union and were able to weight-bear independently or with an assistive device at a mean follow-up of 20.6 months.

The effectiveness of the nail/plate construct has also been well described in the management of femoral non-unions [15]. In the study of Birjandinejad et al. [15], 25 cases of femoral non-unions were all successfully treated with the use of a nail-plate construct and 80% were able to tolerate weight-bearing immediately after the repair.

### The biomechanical concept of nail/plate construct

The nail/plate construct for the management of distal femur fractures includes the placement of a rIMN along with a distal lateral plate [16]. Several techniques have been described and a different combination of implants, with the insertion of the rIMN, usually prior to the use of the distal lateral plate.

The insertion of the rIMN provides an adequate spanning of the whole length of the femur and also provides preliminary stability of the fracture site [17, 18]. The addition of the distal lateral plate provides extra stability and by linking the two implants together, the weight-bearing forces that occur while the patient mobiles are transferred smoothly between the bone and implants [19].

In the biomechanical study of Basci et al. [20], the combination of the nail/plate construct was found to be more resistant to displacement than the lateral plate or rIMN under axial and torsional load, while also the nail/plate construct had the highest number of cycles to failure than the single lateral plate constructs.

The biomechanical superiority of the NP construct has also been underlined in the study of Fontenot et al. [21]. Focusing on the osteoporotic 33C fracture model, the authors conclude that the NP construct required 1.8 the number of cycles to failure and had 100% survivability after maximum loading in comparison to single lateral plating. These findings indicate that the combined use of a nail/plate construct leads to increased axial and torsional stability and allows patients to tolerate full weight bearing immediately postoperatively.

### To link or not the nail/plate construct?

The option of linking the nail/plate construct has been debated and there is no clear evidence whether it leads to superior outcomes. It is believed that by linking the implants together, a more equal distribution of the load between the nail and plate can be achieved, avoiding premature construct failure to one of the devices [17]. In the study of Liporace et al. [22], linking the nail and plate construct is recommended by using two linked screws in severely osteoporotic cases, achieving a more equal distribution of the load between the two devices (Table 2).

**Table 2:** Studies on NP construct for distal femur fractures and non-unions.

Passias et al. [12]	Retrospective cohort, level-1 trauma center	8 cases of NP fixation (97 cases in total)	100% union rate for NP fixation vs. 69% for single fixation group
Kanabur et al. [14]	Retrospective study, NP fixation	8 cases	100% healing rate, NWB for the first 4 weeks
Birjandinejad et al. [15]	Retrospective study on non-unions, 25 femoral non-unions included	25 femoral non-unions	100% union rate for femoral non unions, 80% tolerate weight-bearing immediately after fixation
Basci et al. [20]	Biomechanical analysis	Biomechanical analysis of NP fixation vs. rIMN vs. LP	NP construct has highest number of cycles to failure vs. LP/rIMN
Fontenot et al. [21]	Biomechanical study on 33C fracture model	Biomechanical analysis	NP construct required 1.8 the amount of cycles to failure 100% survivability
Liporace et al. [22]	Retrospective NP fixation	15 cases	14/15 healing rate, 8/15 lost one level of independence

NP: nail/plate fixation, NWB: non weight bearing, rIMN: retrograde intramedullary nailing, LP: lateral femoral plate.

In our study, all cases of distal femur fractures were linked constructs and the interlocking compression bolt (condyle screw) of the T2 retrograde intramedullary nail (Stryker, Minnesota, USA) was used by using the “perfect circle” technique with the fluoroscopy to link the two implants together. From our experience, this technique provides adequate stability to the fracture site, no metalwork failure has been noted and all patients were allowed to mobilize full weight bearing, immediately postoperatively, with no restrictions.

## Conclusions

The optimal management of distal femur fractures, still remains controversial with several different techniques described focusing on early mobilization and adequate fixation. The increasing age population and high activity demands, along with the increased physiological needs of the elderly population lead to an increased need for double fixation constructs that allow early mobilization, resist varus collapse, and provide a synergic environment to stimulate fracture union.

For the management of complex distal femur fractures, the linked nail/plate construct seems to provide adequate stability and high union rates and allows for immediate mobilization. From our cohort study, there was no associated increased complication risk with this technique and the union rate was 100%.

We cannot conclude if there is any significant clinical benefit of linking the nail to the plate. Our results though indicate that the linked nail/plate construct is a valid method that could potentially benefit fracture healing by increasing the sturdiness of the construct, allowing equal distribution of the stress load.

## Data Availability

The data that support the findings of this study are available on request from the corresponding author.
